# Mitochondrial Ca^2+^ Influx via MCU-1 Contributes to Oxidative Mitochondrial Defects in PDR-1/Parkin-Deficient *Caenorhabditis elegans* Body-Wall Muscle

**DOI:** 10.3390/antiox15081043

**Published:** 2026-08-21

**Authors:** Masahiro Kawasumi, Mika Teranishi

**Affiliations:** Graduate School of Life Sciences, Tohoku University, Sendai 980-8577, Japan

**Keywords:** Parkinson’s disease, *Caenorhabditis elegans*, body-wall muscle cells, mitochondrial quality control, mitochondrial reactive oxygen species, mitochondrial membrane potential, mitochondrial calcium influx

## Abstract

Parkinson’s disease (PD) is widely regarded as a disorder of dopaminergic neurons that involves mitochondrial dysfunction, impaired mitophagy, and oxidative stress. However, the nature and significance of skeletal muscle pathology remain unclear. In this study, we used *Caenorhabditis elegans*, which lack muscle stem cells in adulthood, to examine the effects of PDR-1/Parkin deficiency on mitochondrial homeostasis and motor function under conditions where muscle regeneration does not occur. Silencing of *pdr-1* attenuated age-related mitochondrial fragmentation in body-wall muscle cells but was associated with later impairments in locomotor activity and loss of nuclear GFP signals, suggesting progressive muscle cell damage. By day 2 of adulthood, mitochondrial reactive oxygen species (mtROS) levels were elevated in muscle cells subjected to *pdr-1* RNAi, and in the *pdr-1(gk448)* mutant this mtROS elevation was accompanied by a reduction in mitochondrial membrane potential (ΔΨm). In vivo imaging further revealed elevated mitochondrial Ca^2+^ levels ([Ca^2+^]_mito_) in PDR-1-deficient muscle cells. Moreover, the mtROS increase associated with PDR-1 deficiency was suppressed in *mcu-1* mutants. These findings support a model in which MCU-1-dependent elevation of [Ca^2+^]_mito_ contributes to oxidative mitochondrial defects in PDR-1/Parkin-deficient muscle.

## 1. Introduction

Parkinson’s disease (PD) is a neurodegenerative disorder characterized by the degeneration and loss of dopaminergic neurons in the substantia nigra of the midbrain and is a prototypical age-related disease in which mitochondrial dysfunction plays a key role [1,2]. The progressive parkinsonism observed following the loss of functional mitochondrial complex I (MCI) in dopaminergic neurons of the substantia nigra, together with several PD-associated genes and risk factors linked to mitophagy abnormalities, indicates that mitochondrial dysfunction, oxidative stress, and impaired mitophagy are major contributors to PD pathogenesis [3,4,5]. Within mitochondria, calcium (Ca^2+^) acts as a stress signal that can trigger mitophagy when Ca^2+^ accumulation becomes excessive [6]. Mitochondria rapidly take up Ca^2+^ via the mitochondrial Ca^2+^ uniporter (MCU) complex to couple intracellular Ca^2+^ signals to metabolic output; however, dysregulated mitochondrial Ca^2+^ handling can drive excessive ROS production, loss of mitochondrial membrane potential (ΔΨm), and cell death in various disease contexts [7]. Mitochondrial Ca^2+^ overload enhances mitochondrial reactive oxygen species (mtROS) generation and oxidative stress and, when mitophagy is insufficient, promotes the accumulation of damaged mitochondria and ultimately cell death [8]. Substantia nigra dopaminergic neurons exhibit strong cytosolic and mitochondrial Ca^2+^ influx due to their intrinsic pacemaker activity, which has been proposed to enhance mitochondrial oxidative stress and increase susceptibility to degeneration [9]. Importantly, skeletal muscle cells also experience large cytosolic and mitochondrial Ca^2+^ influx during contraction cycles. In the nematode *Caenorhabditis elegans*, which possesses striated muscle structures analogous to human skeletal muscle, aging is associated with an MCU-1-dependent increase in mitochondrial Ca^2+^ concentrations in body-wall muscle, leading to mitochondrial fragmentation and volume loss through mitophagy activity [10]. In the same study, loss of *pdr-1*, the ortholog of human Parkin, was shown to attenuate age-associated mitochondrial fragmentation in body-wall muscle, and the autophagy protein LGG-1, an LC3 homolog, accumulates at sites of fragmented mitochondria, consistent with localized mitophagy activity. These observations suggest that, in *C. elegans* body-wall muscle, mitochondrial Ca^2+^ levels ([Ca^2+^]_mito_) and Parkin-dependent mitophagy pathways jointly shape mitochondrial morphology and contribute to age-associated changes in muscle function.

Motor symptoms of PD, including muscle rigidity, bradykinesia, and postural instability, primarily reflect impaired control of skeletal muscle movement [11,12]. Sarcopenia, an age-related skeletal muscle disorder characterized by loss of muscle mass and strength, is also frequently observed in patients with PD [13]. Although these motor symptoms are often attributed to degeneration of dopaminergic neurons, several studies suggest that intrinsic abnormalities in skeletal muscle may contribute to PD motor deficits. Reports on skeletal muscle mitochondrial function in PD patients are inconsistent: some studies describe preserved mitochondrial function, whereas others report reduced activity of the mitochondrial respiratory chain, including significantly decreased MCI activity in the skeletal muscle of a subset of patients [14,15,16,17,18]. These observations raise the possibility that, at least in a subset of PD patients, primary mitochondrial dysfunction may occur in skeletal muscles in parallel with, or in addition to, neuronal pathology.

The mitophagy pathway mediated by Parkin, an E3 ubiquitin ligase encoded by a causative gene responsible for autosomal recessive juvenile PD, regulates both basal and exercise-induced mitophagy in mature muscle fibers, as well as mitochondrial quality control and fate determination in muscle stem cells [19,20]. Thus, in PD, abnormalities in mitochondrial function and disruptions in Parkin-dependent mitophagy are suggested to occur not only in neurons but also in skeletal muscles. However, it has remained unclear how these alterations in muscle mitochondria contribute to the manifestation and progression of PD motor symptoms, and to what extent mitochondrial Ca^2+^ handling is involved. One difficulty is that, in mammalian models, it is challenging to distinguish between muscle changes secondary to reduced activity caused by dopaminergic neurodegeneration and a primary defect intrinsic to skeletal muscle. In addition, mammalian skeletal muscle contains both mature fibers and muscle stem cells, making it difficult to determine whether changes in mitochondrial quality control primarily affect muscle through atrophy of mature fibers or dysfunction of muscle stem cells. These issues highlight the need for model systems that allow the muscle-intrinsic consequences of Parkin deficiency to be examined in the absence of ongoing muscle regeneration.

To isolate the muscle-intrinsic effects of Parkin dysfunction from secondary consequences of neurodegeneration, we used *C. elegans* as a model system. Adult *C. elegans* completely lack muscle stem cells and cannot regenerate muscle tissue [21]. Therefore, *C. elegans*, whose body-wall muscles are functionally similar to the skeletal muscles of vertebrates, provides an advantageous model in which pathological changes arising specifically in mature muscle fibers can be readily detected. Furthermore, in *C. elegans*, it is well established that RNA interference (RNAi) administered through feeding in a wild-type (WT) background generally exerts minimal effects on neurons, because systemic RNAi depends on SID-1–mediated dsRNA uptake and many neurons are largely refractory to this pathway [22,23]. Under these conditions, feeding RNAi primarily suppresses gene function in body-wall muscle and thus provides a practical means to assess how PD-related factors may act within muscle cells, even though the possibility of minor neuronal effects cannot be entirely excluded.

In *C. elegans*, *pdr-1* is the sole ortholog of human Parkin and mediates mitochondrial quality control through conserved pathways [24]. Given that Parkin mutations are a major cause of autosomal recessive early onset Parkinson’s disease and that skeletal muscle dysfunction is a prominent feature in patients with PD, understanding mitochondrial pathology in muscle may provide insights into non-neuronal manifestations of the disease. In this study, we investigated how PDR-1/Parkin deficiency affects mitochondrial function and locomotor activity in the body-wall muscle cells of *C. elegans* and examined whether the mitochondrial defects are associated with mitochondrial Ca^2+^ influx. Our study showed that PDR-1 deficiency increased mtROS and reduced ΔΨm in body-wall muscle cells. These changes were suppressed when mitochondrial Ca^2+^ influx via MCU-1 was inhibited, indicating that MCU-1-dependent Ca^2+^ entry is required for the Ca^2+^-dependent component of this mitochondrial dysfunction.

## 2. Materials and Methods

### 2.1. C. elegans Strains and Culture Conditions

We maintained *C. elegans* strains using standard methods [25]. All strains were cultured on nematode growth medium (NGM) plates with OP50 as a food source at 20 °C. To obtain age-synchronized populations, adult hermaphrodites were placed on fresh NGM plates and allowed to lay eggs for 3 h, after which the adults were removed. The eggs were then cultured at 20 °C, and L4-stage worms were picked and designated as day 0. All experiments were performed using age-matched animals. The following strains were used in this study: N2 (WT), VC1024: *pdr-1(gk448)*, CZ19982: *mcu-1(ju1154)*, SD1347: *ccIs4251 [myo-3p::GFP::LacZ::NLS + myo-3p::mitochondrial GFP]*, ATU3301: *ccIs4251 [myo-3p::GFP::LacZ::NLS + myo-3p::mitochondrial GFP] aceIs1 [Pmyo-3::mitochondrial LAR-GECO + Pmyo2::RFP]*, and ATU3302: *pdr-1(gk448) ccIs4251 [myo-3p::GFP::LacZ::NLS + myo-3p::mitochondrial GFP] aceIs1 [Pmyo-3::mitochondrial LAR-GECO + Pmyo2::RFP]*.

### 2.2. RNAi Treatment

**Figure 1:**
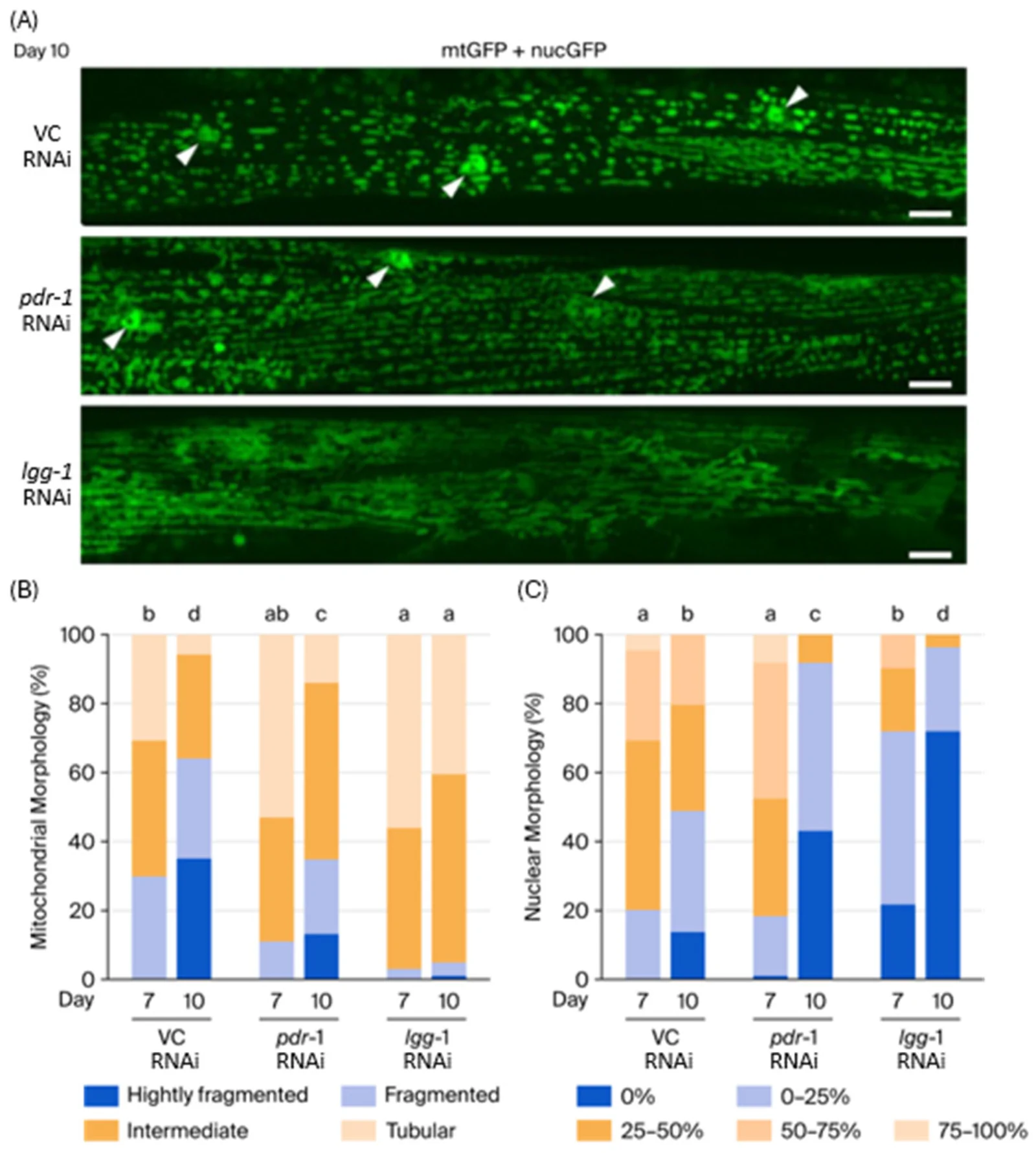
RNAi-mediated silencing of *pdr-1* and *lgg-1* mitigates age-related mitochondrial fragmentation and promotes nuclear degradation in *C. elegans* muscle cells. (**A**) Representative fluorescence images of body-wall muscle cells in transgenic *C. elegans* expressing mitochondrion-targeted GFP (mtGFP) and nucleus-targeted GFP (nucGFP) on day 10 of adulthood. Arrowheads indicate nuclei. Scale bars: 10 µm. (**B**) Quantitative analysis of mitochondrial morphology in body-wall muscle cells. Mitochondrial morphology was classified into four categories as described in the Materials and Methods. Blue, light blue, orange, and light orange indicate the proportions of cells exhibiting highly fragmented, fragmented, intermediate, and tubular mitochondrial morphologies, respectively. (**C**) Quantitative analysis of nuclear morphology in body-wall muscle cells. Blue, light blue, orange, light orange, and pale orange indicate the proportions of cells displaying nuclear GFP fluorescence at 0%, 0–25%, 25–50%, 50–75%, and 75–100%, respectively. The data represent *n* = 53–57 cells per condition, obtained from at least 12 animals across three independent experiments. Different letters above the bars indicate statistically significant differences (chi-square test, *p* < 0.05).

**Figure 2:**
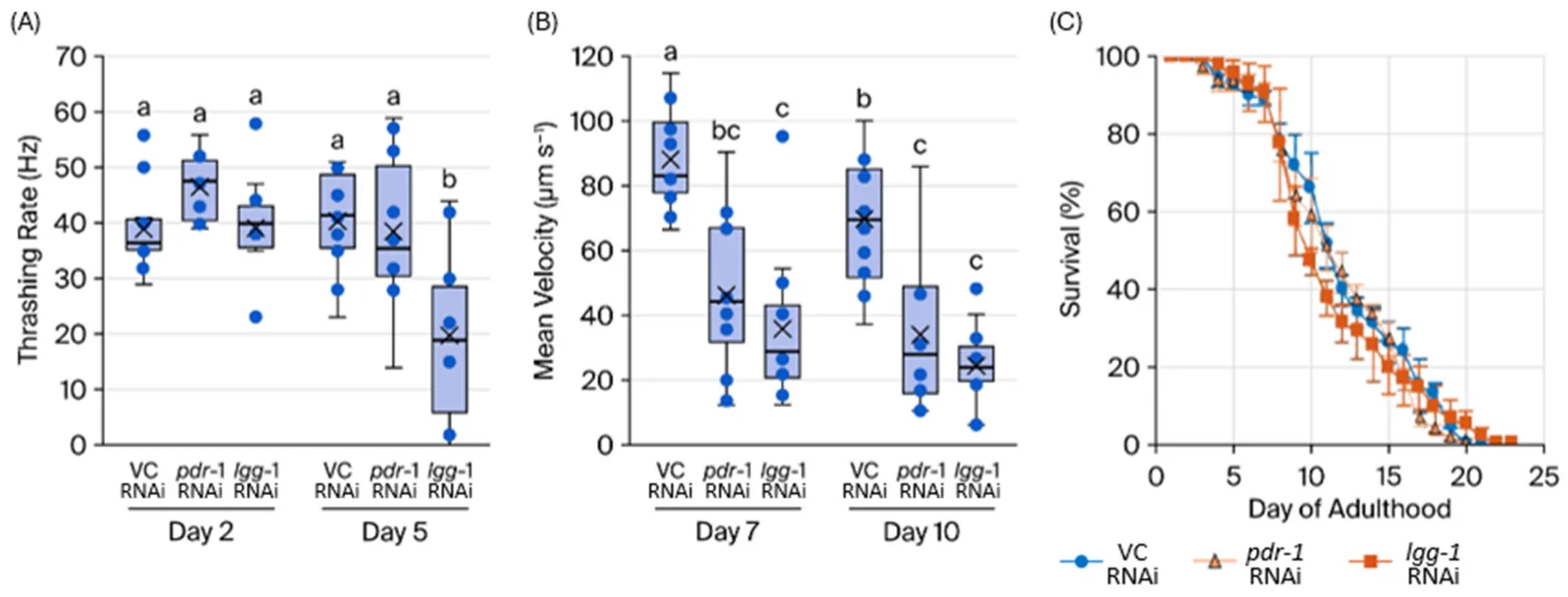
RNAi-mediated silencing of *pdr-1* and *lgg-1* reduces locomotor activity but does not affect lifespan in aging *C. elegans*. (**A**) Thrashing rate (Hz) in vector control (VC), *pdr-1* RNAi, and *lgg-1* RNAi animals on days 2 and 5 of adulthood. Box-and-whisker plots show the distribution of values, and individual dots represent single animals (*n* = 10 per condition). (**B**) Mean locomotion velocity of VC, *pdr-1* RNAi, and *lgg-1* RNAi animals on days 7 and 10 of adulthood. Box-and-whisker plots show the distribution of values, and individual dots represent single animals (*n* = 12 per condition). (**C**) Survival curves of VC, *pdr-1* RNAi, and *lgg-1* RNAi animals. The data are presented as percent survival (mean ± standard error) from four independent experiments, with 25 to 30 worms per experiment. Different letters above the bars indicate statistically significant differences (Tukey’s multiple comparison test, *p* < 0.05).

For *lgg-1* and *mcu-1* RNAi, we used clones from the Ahringer RNAi feeding library (Source BioScience, Nottingham, UK; clone C32D5.9 and K02B2.3, respectively). For *pdr-1* RNAi, a fragment amplified from genomic DNA using the *pdr-1* RNAi Fw and Rv primers (Table S1) was cloned into the L4440 vector digested with HindIII and XbaI. RNAi was performed via bacterial feeding as described by [26]. For the experiments shown in Figures 1, 2, 4 and 5, RNAi treatment was initiated at the L4 stage. For the experiments shown in Figure 6, RNAi treatment was initiated either at the L4 stage or on day 7 of adulthood, as indicated. For RNAi treatment initiated on day 7 of adulthood, worms were maintained on VC RNAi plates until day 7 of adulthood and were then transferred to plates seeded with bacteria expressing *mcu-1* dsRNA. Worms of the indicated strains were transferred to NGM plates seeded with HT115(DE3) bacteria expressing the corresponding dsRNA. Bacteria containing an empty L4440 vector were used as the vector control (VC). Worms were transferred to freshly prepared RNAi plates every two days.

### 2.3. Imaging of Mitochondrial Morphology and Nuclear Morphology

Mitochondrial and nuclear morphology in body-wall muscle cells was analyzed by assaying the expression of the transgene *ccIs4251 [myo-3p::GFP::LacZ::NLS + myo-3p::mitochondrial GFP]*, in which the GFP signal is localized to the mitochondrial matrix in body-wall muscle cells [27,28]. Synchronized worms were mounted on a microscope slide with a 6.5 mm square, 20 μm deep well made with a water-repellent coating (Matsunami Glass Ind., Ltd., Osaka, Japan). Worms were immobilized with 100 mM sodium azide. GFP fluorescence images were obtained using an FV10i confocal laser-scanning microscope (Olympus Co., Tokyo, Japan).

Mitochondria were classified into four morphological categories according to Regmi et al. [29] as follows: “tubular,” comprising images showing a majority of long interconnected mitochondrial networks; “intermediate,” consisting of images showing a combination of interconnected mitochondrial networks and smaller fragmented mitochondria; “fragmented,” consisting of images showing a majority of short mitochondria; and “highly fragmented,” comprising images showing sparse small round mitochondria (Figure S1A). The residual level of nuclear GFP fluorescence was scored semi-quantitatively by visual inspection of nuclei using a five-grade scale: 0%, 0–25%, 25–50%, 50–75%, and 75–100% of nuclei showing GFP fluorescence (Figure S1B).

### 2.4. Measurement of Locomotion Activity and Mean Velocity

We recorded the movements using a stereomicroscope (SMZ18; Nikon, Tokyo, Japan), a digital camera (DP74; Olympus), and imaging software (cellSens Standard 2.2; Olympus). Synchronized worms were transferred to an NGM liquid medium to assess locomotor activity. Locomotor activity was quantified as the average thrashing rate after gently tapping the plate to induce movement. Thrashes were counted for 1 min per worm. To determine the moving velocity, synchronized worms were transferred to NGM plates without bacteria. The distance moved every 5 s was measured, and the mean of four consecutive measurements was calculated for each worm.

### 2.5. Measurement of mtROS, ΔΨm and [Ca2+]_mito_ in Body-Wall Muscle Cells

mtROS levels in body-wall muscle cells were measured using MitoTracker™ Red CM-H_2_Xros (M7513, Thermo Fisher Scientific, Waltham, MA, USA). A staining medium was prepared by mixing MitoTracker™ Red CM-H_2_Xros at a final concentration of 10 µM with OP50 bacteria whose optical density at 600 nm (OD_600_) was adjusted to 0.1 in NGM liquid medium. For RNAi experiments, HT115(DE3) bacteria harboring the respective RNAi clones were used instead of OP50, and their OD_600_ was adjusted to 0.1. Worms were transferred to the staining medium and incubated for two days before imaging. Prior to imaging, the worms were washed with M9 buffer for 30 min under light-protected conditions.

To measure ΔΨm, tetramethylrhodamine ethyl ester (TMRE; MedChemExpress, Monmouth Junction, NJ, USA) was added at a final concentration of 8 µM, and the worms were stained for 6 h. Prior to imaging, the worms were washed with M9 buffer for 30 min under light-protected conditions.

[Ca^2+^]_mito_ in body-wall muscle cells were measured using a mitochondrion-targeted red fluorescent Ca^2+^ indicator (mito-LAR-GECO1.2) [10].

**Figure 3:**
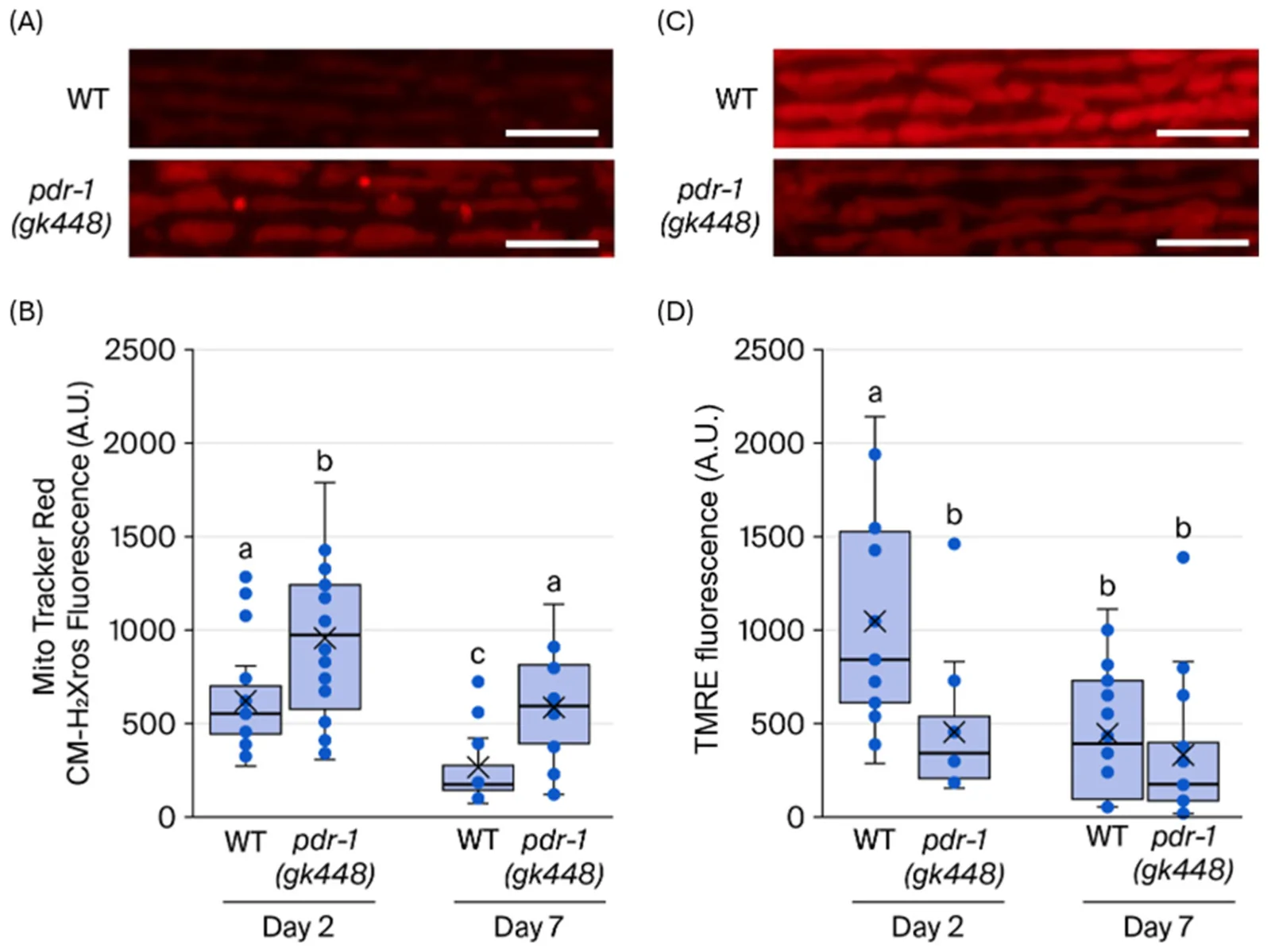
The *pdr-1* mutation increases mitochondrial reactive oxygen species (mtROS) levels and reduces mitochondrial membrane potential (ΔΨm) in *C. elegans* muscle cells. (**A**) Representative fluorescence images of MitoTracker^TM^ Red CM-H2Xros in body-wall muscle cells of N2 wild-type (WT) and *pdr-1(gk448) C. elegans* on day 2 of adulthood. Scale bars: 5 µm. (**B**) Quantification of mtROS fluorescence intensity in WT and *pdr-1(gk448)* animals on days 2 and 7 of adulthood. Fluorescence intensity is expressed in arbitrary units (A.U.) and was measured as the mean pixel intensity within manually defined regions of interest after background subtraction. Data are shown as box-and-whisker plots, with individual dots representing single cells (*n* = 20–27 cells per condition), obtained from at least five animals per group. (**C**) Representative fluorescence images of tetramethylrhodamine ethyl ester (TMRE) in body-wall muscle cells of WT and *pdr-1(gk448) C. elegans* on day 2 of adulthood. Scale bars: 5 µm. (**D**) Quantification of TMRE fluorescence intensity in WT and *pdr-1(gk448)* animals on days 2 and 7 of adulthood. Fluorescence intensity (A.U.) was quantified as in (B). Data are shown as box-and-whisker plots, with individual dots representing single cells (*n* = 18–21 cells per condition), obtained from at least five animals per group. Different letters above the bars indicate statistically significant differences (Tukey’s multiple comparison test, *p* < 0.05).

**Figure 4:**
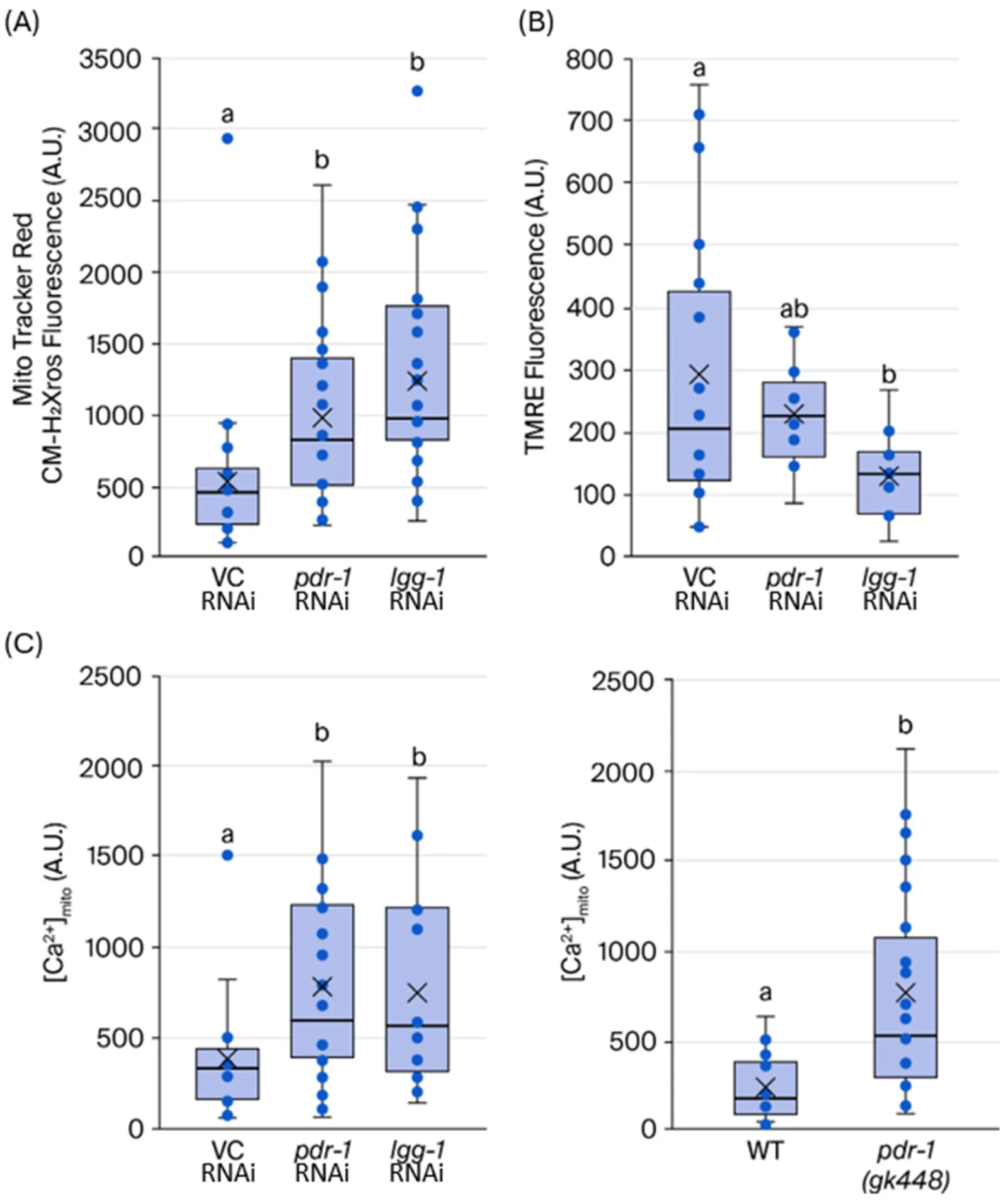
Loss of *pdr-1* or *lgg-1* function elevates mtROS and mitochondrial Ca^2+^ levels ([Ca^2+^]_mito_), with a reduction in ΔΨm following *lgg-1* silencing, in *C. elegans* muscle cells. (**A**) Quantification of mtROS fluorescence intensity in VC, *pdr-1* RNAi, and *lgg-1* RNAi animals on day 2 of adulthood. (**B**) Quantification of TMRE fluorescence intensity in VC, *pdr-1* RNAi, and *lgg-1* RNAi animals on day 2 of adulthood. (C) Quantification of [Ca^2+^]_mito_ in VC, *pdr-1* RNAi, and *lgg-1* RNAi animals (left) and WT and *pdr-1(gk448)* animals (right) on day 2 of adulthood. For (**A**–**C**), fluorescence intensity (A.U.) was measured as the mean pixel intensity within manually defined regions of interest after background subtraction. Data are shown as box-and-whisker plots, with individual dots representing single cells (*n* = 28–32, 12–18, and 20–22 cells per condition for (**A**), (**B**), and (**C**), respectively), obtained from at least five animals per group. Different letters above the bars indicate statistically significant differences (one-way ANOVA followed by Tukey’s multiple comparison test, *p* < 0.05).

**Figure 5:**
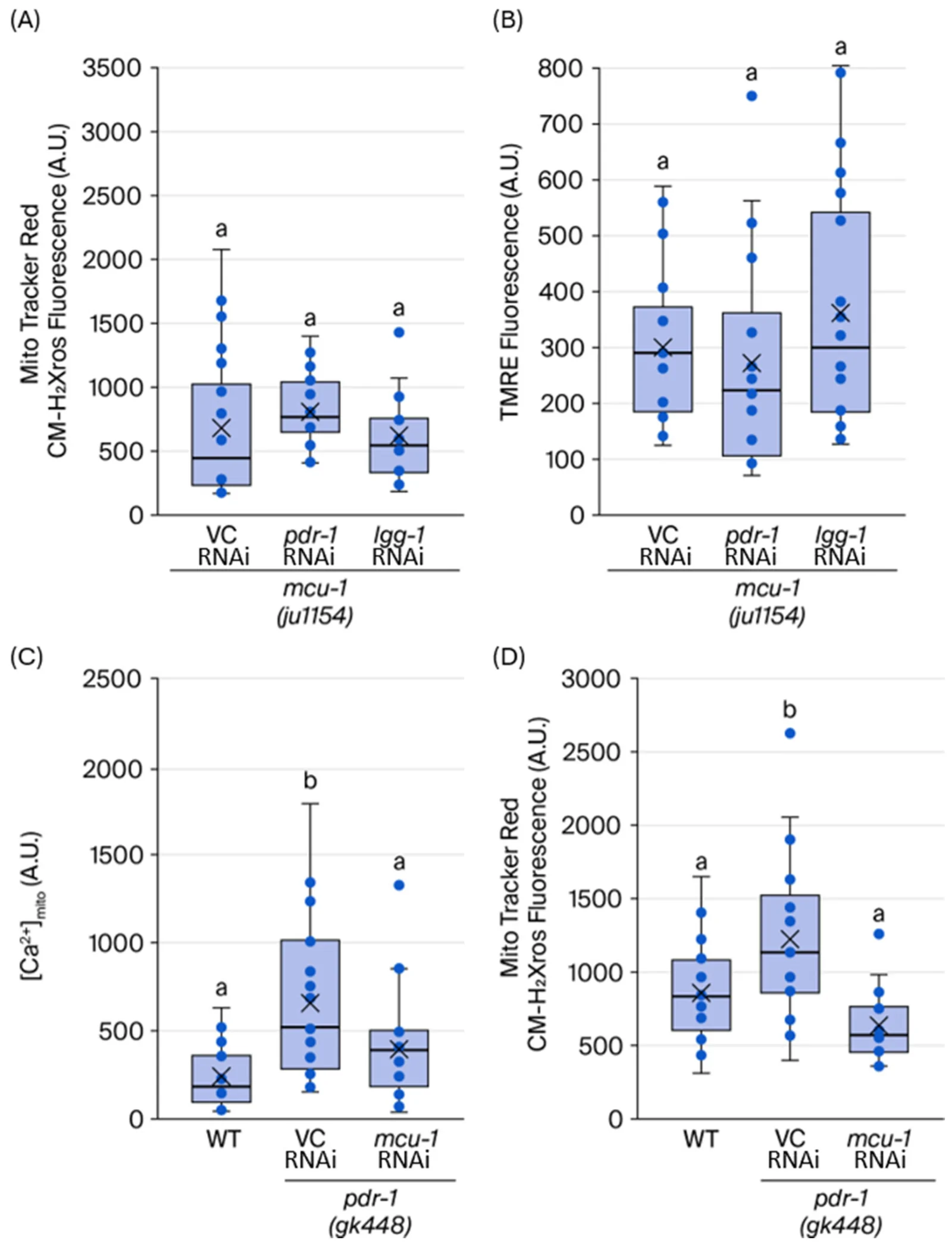
The *mcu-1* mutation suppresses mitochondrial dysfunction associated with loss of *pdr-1* and *lgg-1* function in *C. elegans* muscle cells. (**A**) Quantification of mtROS fluorescence intensity in *mcu-1(ju1154)* mutants after 2 days of RNAi treatment (day 2 of adulthood). (**B**) Quantification of TMRE fluorescence intensity in *mcu-1(ju1154)* mutant after 2 days of RNAi treatment (day 2 of adulthood). (**C**) Quantification of [Ca^2+^]_mito_ in WT, VC, and *mcu-1* RNAi animals on day 2 of adulthood. WT data in (**C**) are the same as those shown in Figure 4C. (**D**) Quantification of mtROS fluorescence intensity in WT, VC, and *mcu-1* RNAi animals on day 2 of adulthood. For (**A**–**D**), fluorescence intensity (A.U.) was measured as the mean pixel intensity within manually defined regions of interest after background subtraction. Data are shown as box-and-whisker plots with individual dots representing single cells. For (**A**,**B**), *n* = 20 cells per condition; for (**C**,**D**), *n* = 18–22 cells per condition, obtained from at least five animals per group. Different letters above the bars indicate statistically significant differences (one-way ANOVA followed by Tukey’s multiple comparison test, *p* < 0.05).

**Figure 6:**
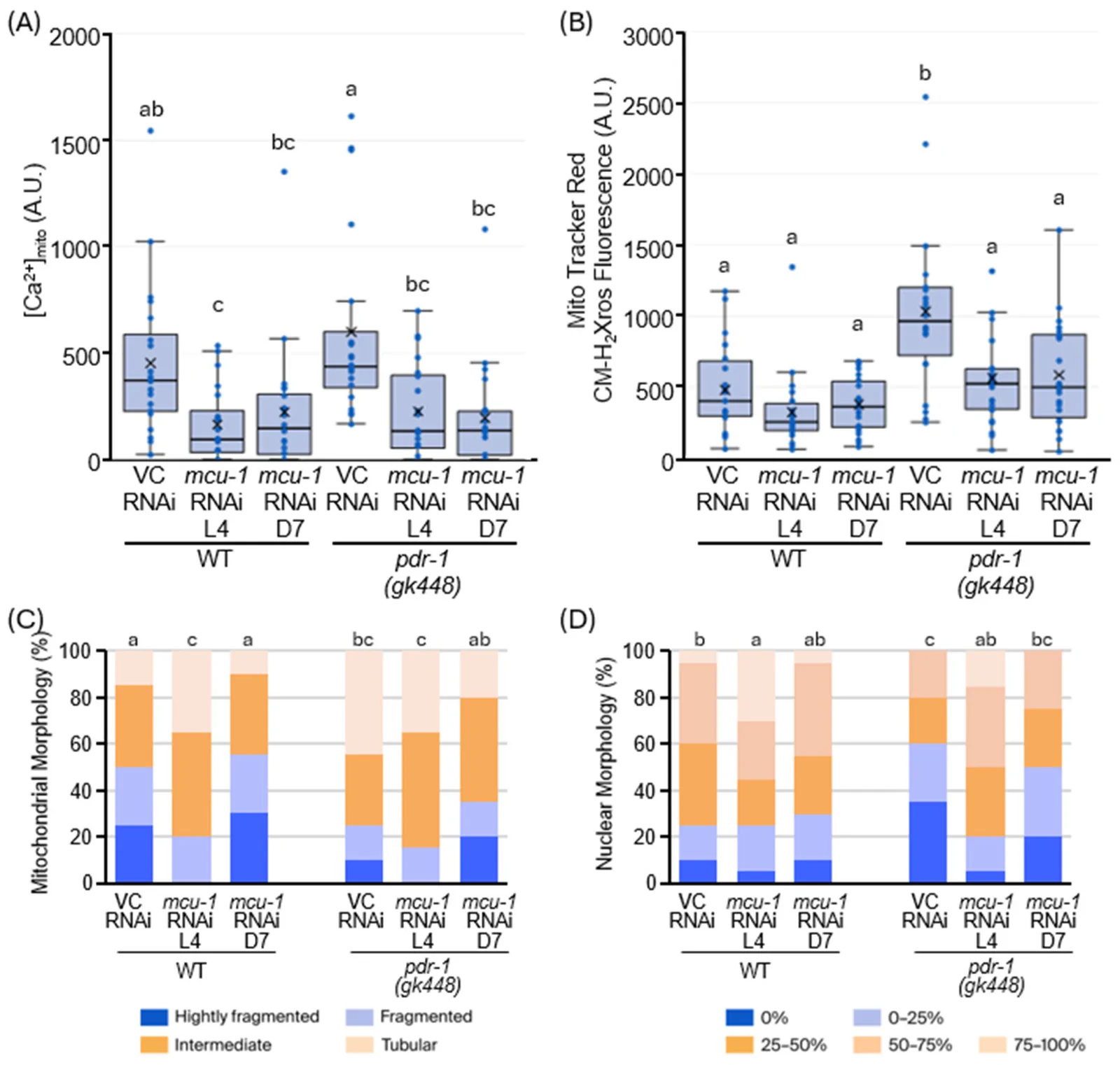
The timing of *mcu-1* RNAi differentially affects [Ca^2+^]_mito_, mtROS, mitochondrial morphology, and nuclear degradation in aging *C. elegans* muscle cells. (**A**,**B**) Quantification of [Ca^2+^]_mito_ (**A**) and mtROS fluorescence intensity (**B**) in WT and *pdr-1(gk448)* animals on day 10 of adulthood. Animals were treated with VC RNAi, *mcu-1* RNAi initiated at the L4 stage, or *mcu-1* RNAi initiated on day 7 of adulthood. For (**A**,**B**), fluorescence intensity (A.U.) was measured as the mean pixel intensity within manually defined regions of interest after background subtraction. Because images for (**A**,**B**) were acquired using microscope settings different from those used for Figure 3, Figure 4 and Figure 5, fluorescence intensity values are not directly comparable between these figures. Data are shown as box-and-whisker plots with individual dots representing single cells. (**C**,**D**) Quantitative analysis of mitochondrial morphology (**C**) and nuclear morphology (**D**) in body-wall muscle cells of WT and *pdr-1(gk448)* animals on day 10 of adulthood. Animals were treated with VC RNAi, *mcu-1* RNAi initiated at the L4 stage, or *mcu-1* RNAi initiated on day 7 of adulthood. Mitochondrial and nuclear morphologies were classified as described for Figure 1. For (**A**,**B**), *n* = 20–22 cells per condition; for (**C**,**D**), *n* = 40 cells per condition, obtained from at least 10 animals per group. Different letters indicate statistically significant differences (one-way ANOVA followed by Tukey’s multiple comparison test for (**A**,**B**) and chi-square test for (**C**,**D**), *p* < 0.05).

For all measurements presented in Figures 3, 4 and 5, fluorescence images were acquired using identical microscope settings across all genotypes within each experiment. For the experiment presented in Figure 6, which used aged animals, microscope settings were adjusted as appropriate for image acquisition; however, identical settings were applied across all genotypes within that experiment. Image acquisition and quantification were performed using the dedicated FV10-ASW software (ver4.2a) provided with the FV10i confocal laser-scanning microscope (Olympus Co.). Fluorescence intensity was quantified as the mean pixel intensity within manually defined regions of interest after background subtraction.

### 2.6. Real-Time Quantitative RT-PCR

Total RNA was extracted from 20 synchronized worms using a TRI reagent (Molecular Research Center, Cincinnati, OH, USA). cDNA was synthesized using a PrimeScript RT Reagent Kit with a gDNA Eraser (Takara Bio Inc., Shiga, Japan). Real-time quantitative RT-PCR was performed using the primer sets listed in Table S1. The data were normalized to the expression of *act-1*. The experiment was repeated six times using independent biological replicates.

### 2.7. Statistical Analysis

We performed statistical analyses using R (version 4.4.2). One-way ANOVA followed by Tukey’s multiple-comparisons test was used for comparisons involving more than two groups. Categorical data were analyzed by chi-square tests, and *p*-values were corrected for multiple comparisons using the Holm–Bonferroni method. Sample sizes and the number of independent experiments are indicated in the figure legends.

## 3. Results

### 3.1. Silencing of pdr-1 and lgg-1 Mitigates Age-Related Mitochondrial Fragmentation but Promotes Nuclear Degradation

In this study, we used RNAi to suppress the expression of *pdr-1* and *lgg-1* in *C. elegans* body-wall muscle and examined the resulting mitochondrial and nuclear phenotypes. LGG-1, the *C. elegans* homolog of mammalian LC3, localizes to autophagosomal membranes and participates in both Parkin-dependent and Parkin-independent mitophagy, as well as in the selective autophagy of other organelles [30,31]. To assess the relative contribution of the PDR-1 pathway, we compared the effects of *pdr-1* knockdown with those of *lgg-1* knockdown. Mitochondrial morphology in body-wall muscle cells was visualized using mitochondrion-targeted GFP at day 10 after the L4 stage (Figure 1A). In *pdr-1* RNAi animals, age-related mitochondrial fragmentation was attenuated compared with animals treated with the empty vector control (VC). In contrast, *lgg-1* RNAi animals exhibited a more extensively interconnected tubular mitochondrial network than *pdr-1* RNAi animals.

Quantitative analysis of age-associated mitochondrial morphology from day 7 to day 10 showed that mitochondrial fragmentation in VC animals was more advanced on day 10 than on day 7 (Figure 1B) [10]. In *pdr-1* RNAi animals, mitochondrial fragmentation on day 7 did not differ significantly from that in age-matched VC animals; however, by day 10, mitochondria were significantly less fragmented than in VC. In *lgg-1* RNAi animals, mitochondrial fragmentation was significantly suppressed relative to that in the VC at both time points.

Nucleus-targeted GFP serves as a marker for assessing cell survival and death [32]. Therefore, we examined nuclear GFP fluorescence under each RNAi condition. While nuclear GFP signals were detected in both VC and *pdr-1* RNAi animals, no nuclear GFP signals were observed in *lgg-1* RNAi animals (Figure 1A). Quantitative analysis revealed that there was no significant difference in nuclear GFP fluorescence between *pdr-1* RNAi and VC animals on day 7 (Figure 1C). However, on day 10, nuclear degradation was more evident in *pdr-1* RNAi animals. In contrast, *lgg-1* RNAi animals showed a significantly greater loss of nuclear GFP fluorescence at both ages, indicating the progression of cell death.

### 3.2. Silencing of pdr-1 and lgg-1 Reduces Locomotor Activity During Aging Without Affecting Lifespan in C. elegans

To examine whether nuclear degradation in body-wall muscle cells was associated with functional decline, we next determined the impact of *pdr-1* and *lgg-1* silencing on locomotor activity in *C. elegans*, we evaluated their movement behavior at different ages. On day 2, the thrashing rates of *pdr-1* and *lgg-1* RNAi-treated animals did not differ significantly from those of VC animals (Figure 2A). However, by day 5, the thrashing rate of *lgg-1* RNAi animals was significantly lower than that of the VC. Locomotion velocity on days 7 and 10 of adulthood was significantly reduced in both *pdr-1* and *lgg-1* RNAi animals compared to VC animals (Figure 2B). These results suggest that while *lgg-1* silencing accelerates the decline in locomotor activity, *pdr-1* silencing primarily impairs this activity at older ages.

Next, we investigated whether RNAi treatment of *pdr-1* and *lgg-1* affected lifespan. Neither *pdr-1* nor *lgg-1* RNAi caused any significant changes in lifespan compared to VC animals (Figure 2C). This suggests that silencing these genes does not significantly affect the longevity of *C. elegans*.

### 3.3. The pdr-1 Mutation Elevates mtROS and Diminishes ΔΨm

Next, we investigated mtROS levels in *pdr-1* mutants. On day 2 of adulthood, the *pdr-1(gk448)* mutant exhibited higher mtROS fluorescence intensity than WT animals (Figure 3A). Quantitative analysis confirmed that ROS fluorescence intensity on day 2 of adulthood was significantly elevated in *pdr-1(gk448)* compared to that in WT animals (Figure 3B), and this difference persisted on day 7 of adulthood.

We then examined ΔΨm by measuring TMRE fluorescence intensity. On day 2 of adulthood, TMRE intensity decreased in *pdr-1(gk448)* mutants, indicating a reduction in ΔΨm (Figure 3C). Quantitative analysis showed that ΔΨm on day 2 was significantly lower in *pdr-1(gk448)* animals than in WT animals (Figure 3D). By day 7 of adulthood, ΔΨm was reduced in WT animals, such that there was no longer a significant difference between WT and *pdr-1(gk448)*. These results indicate that in *pdr-1(gk448)* animals, mtROS levels are elevated and ΔΨm is reduced by day 2 of adulthood.

### 3.4. Silencing of pdr-1 and lgg-1 Induces Mitochondrial Dysfunction Accompanied by Elevated [Ca2+]_mito_

In *pdr-1(gk448)*, *pdr-1* is genetically disrupted and therefore absent throughout development. Next, we examined whether similar mitochondrial alterations could be induced when *pdr-1* or *lgg-1* was silenced during the larval-to-adult transition. RNAi was initiated at the L4 stage, and mtROS levels and ΔΨm were measured two days later, on day 2 of adulthood (Figure 4A,B). A significant increase in mtROS was observed in *pdr-1* RNAi (Figure 4A). In *lgg-1* RNAi, a significant increase in mtROS was observed compared to VC animals, although no significant difference was observed between *pdr-1* and *lgg-1* RNAi animals. When ΔΨm was measured, no significant difference was observed between *pdr-1* RNAi and VC animals, whereas ΔΨm was significantly reduced in *lgg-1* RNAi animals (Figure 4B).

ROS production is known to be closely linked to mitochondrial Ca^2+^ homeostasis [33]. We examined whether [Ca^2+^]_mito_ were altered under these conditions. We used genetically modified WT worms that expressed a mitochondrion-targeted red fluorescent Ca^2+^ indicator (mito-LAR-GECO1.2) in body-wall muscle cells [10]. [Ca^2+^]_mito_ were significantly elevated in *pdr-1* RNAi and *lgg-1* RNAi animals compared to VC on day 2 of adulthood (Figure 4C). A similar elevation in [Ca^2+^]_mito_ was observed in *pdr-1(gk448)* mutants, consistent with the RNAi results. These findings indicate that impairment of mitophagy-related pathways is associated with increased [Ca^2+^]_mito_ in body-wall muscle cells.

### 3.5. MCU-1 Is Required for Mitochondrial Dysfunction Induced by pdr-1 and lgg-1 Silencing

MCU-1, the mitochondrial calcium uniporter, is the primary channel responsible for mitochondrial Ca^2+^ uptake in *C. elegans* body-wall muscle cells [10]. To investigate whether the observed elevation in [Ca^2+^]_mito_ depended on MCU-1 function, we examined the effects of *pdr-1* and *lgg-1* silencing in *mcu-1(ju1154)* mutants, which lack functional MCU-1. In the *mcu-1(ju1154)* mutant background, RNAi-mediated silencing of *pdr-1* or *lgg-1* did not increase mtROS or reduce ΔΨm (Figure 5A,B). These results indicate that the MCU-1-dependent micochondrial Ca^2+^ influx is required for the increases in mtROS and the reductions in ΔΨm induced by *pdr-1* or *lgg-1* silencing.

We next examined how reducing MCU-1 function modifies [Ca^2+^]_mito_ and mtROS changes caused by PDR-1 deficiency. In *pdr-1(gk448)* mutants, *mcu-1* RNAi resulted in a significant reduction in [Ca^2+^]_mito_ compared to VC animals (Figure 5C), and this was accompanied by a marked suppression of mtROS accumulation (Figure 5D). Both *mcu-1* mutants treated with *pdr-1* RNAi and *pdr-1* mutants treated with *mcu-1* RNAi showed suppression of mtROS elevation (Figure 5A,D). These epistatic interactions suggest a functional interdependence between mitophagy pathways and MCU-1-dependent Ca^2+^ homeostasis.

To exclude the possibility of compensatory transcriptional changes, we examined the expression levels of *pdr-1*, *lgg-1*, and *mcu-1* via real-time quantitative RT-PCR. The *mcu-1* mutation did not alter the expression of *pdr-1* or *lgg-1* (Figure S2A). Similarly, the *pdr-1* mutation did not affect *mcu-1* expression (Figure S2B). These results indicate that the observed genetic interactions are unlikely due to compensatory changes in gene expression.

### 3.6. The Timing of mcu-1 RNAi Differentially Affects Mitochondrial Fragmentation and Nuclear Degradation During Aging

To determine whether MCU-1-dependent Ca^2+^ influx contributes to mitochondrial alterations at a later stage of adulthood, we next examined the effects of *mcu-1* RNAi initiated either at the L4 stage or on day 7 of adulthood in WT and *pdr-1(gk448)* animals at day 10 of adulthood. *mcu-1* RNAi initiated at the L4 stage significantly reduced [Ca^2+^]_mito_ compared with VC RNAi in both WT and *pdr-1(gk448)* animals (Figure 6A). When *mcu-1* RNAi was initiated on day 7 of adulthood, [Ca^2+^]_mito_ showed an insignificant decrease in WT animals relative to VC RNAi, although this difference was not statistically significant, whereas a significant reduction was observed in *pdr-1(gk448)* animals. We next measured mtROS levels in WT and *pdr-1(gk448)* animals (Figure 6B). In WT animals, *mcu-1* RNAi initiated at the L4 stage showed a trend toward reduced mtROS levels relative to VC RNAi; however, this reduction was not statistically significant. In contrast, *mcu-1* RNAi initiated on day 7 of adulthood did not show an apparent reduction in mtROS levels in WT animals. In *pdr-1(gk448)* animals, *mcu-1* RNAi initiated at either the L4 stage or on day 7 of adulthood significantly reduced mtROS levels relative to VC RNAi.

We next examined age-associated mitochondrial morphology and nuclear GFP fluorescence in body-wall muscle cells under these conditions (Figure 6C,D, Figure S3). In WT animals, *mcu-1* RNAi initiated at the L4 stage significantly suppressed mitochondrial fragmentation and preserved nuclear GFP fluorescence, indicating attenuated nuclear degradation. In contrast, *mcu-1* RNAi initiated on day 7 of adulthood did not significantly affect either mitochondrial fragmentation or nuclear GFP fluorescence. In *pdr-1(gk448)* animals, *mcu-1* RNAi did not significantly alter mitochondrial fragmentation; however, when initiated at the L4 stage, *mcu-1* RNAi significantly preserved nuclear GFP fluorescence, indicating suppression of nuclear degradation.

## 4. Discussion

In this study, we examined how PDR-1 deficiency affects mitochondrial properties and locomotor activity in *C. elegans* body-wall muscles. Both genetic disruption of *pdr-1* and RNAi-mediated suppression of *pdr-1* led to elevated mtROS in body-wall muscle early in adulthood. In both contexts, *pdr-1* deficiency was associated with increased [Ca^2+^]_mito_, and inhibition of mitochondrial Ca^2+^ influx via MCU-1 prevented the accompanying mtROS elevation. The epistatic relationship between *mcu-1* and *pdr-1* mutations further suggests that mitophagy pathways and MCU-1-mediated Ca^2+^ homeostasis are functionally coupled.

We found that the lifespan of animals subjected to *pdr-1* or *lgg-1* RNAi did not differ significantly from that of control animals (Figure 2C). This observation indicates that impaired mitophagy does not necessarily shorten lifespan under our experimental conditions, despite clear mitochondrial dysfunction and a decline in locomotion activity. This is also consistent with previous reports indicating that the lifespan of *pdr-1* mutants is comparable to that of WT animals [34]. In *lgg-1* RNAi animals, survival could still be confirmed because they retained head and pharyngeal movements despite having almost completely lost locomotor activity.

mtROS production is known to be closely coupled to ΔΨm. In human cells, it has been shown that selectively increasing mtROS leads to subsequent mitochondrial membrane depolarization [35,36]. In cardiac myocytes, the gradual accumulation of mtROS has been reported to reach a threshold at which the mitochondrial permeability transition is induced, resulting in an abrupt loss of ΔΨm [37]. Collectively, these findings support a functional link between mtROS elevation and mitochondrial depolarization. We observed that *pdr-1* loss-of-function mutants exhibited both increased mtROS and reduced ΔΨm (Figure 3B,D), whereas *pdr-1* RNAi increased mtROS without a detectable decrease in ΔΨm (Figure 4A,B). This is consistent with the notion that RNAi typically induces only a partial loss of function, suggesting that the magnitude or duration of mtROS elevation under *pdr-1* RNAi is likely insufficient to cause measurable depolarization. This contrasts with the more pronounced mitochondrial depolarization observed when autophagosome formation mediated by LGG-1 is impaired. In animals subjected to *lgg-1* RNAi, an increase in mtROS and a decrease in ΔΨm were observed (Figure 4A,B). This finding suggests that a defect in autophagosome formation mediated by LGG-1 permits the accumulation of severely damaged mitochondria, leading to mitochondrial depolarization that is not induced by partial inhibition of PDR-1-mediated mitophagy. Given that LGG-1 acts downstream of PDR-1 and is required not only for Parkin-dependent and Parkin-independent mitophagy but also for broader autophagic processes, these results imply that a broad blockade of autophagic clearance has a more direct and pronounced impact on ΔΨm than selective disruption of PDR-1-dependent mitophagy.

Mitochondrial Ca^2+^ uptake stimulates Ca^2+^-sensitive dehydrogenases in the tricarboxylic acid (TCA) cycle, thereby enhancing oxidative phosphorylation. Under the conditions of high respiratory activity, this Ca^2+^-driven increase in mitochondrial metabolism can promote ROS generation in the respiratory chain [33]. Our findings indicate that MCU-1-dependent mitochondrial Ca^2+^ uptake is required for the [Ca^2+^]_mito_ elevation and mtROS changes that contribute to mitochondrial dysfunction in PDR-1-deficient muscle. However, the present study does not yet identify the upstream source of the Ca^2+^ influx that increases [Ca^2+^]_mito_. Previous studies on human neuroblastoma cells have shown that Parkin deficiency enhances phospholipase C activity, resulting in excessive inositol 1,4,5-trisphosphate (IP_3_)-dependent Ca^2+^ release from the endoplasmic reticulum (ER) and disruption of intracellular Ca^2+^ homeostasis [38]. Given that [Ca^2+^]_mito_ closely follow cytosolic Ca^2+^ transients in live *C. elegans* body-wall muscle cells [10], increased cytosolic Ca^2+^ levels are expected to drive elevated mitochondrial Ca^2+^. Furthermore, in Parkin-deficient models, ER–mitochondria contacts and ER-to-mitochondrial Ca^2+^ transfer are altered, often resulting in increased mitochondrial Ca^2+^ loading during receptor-driven Ca^2+^ signaling [39,40,41]. Taken together, these observations raise the possibility that PDR-1 deficiency may increase [Ca^2+^]_mito_ in muscle through IP_3_-dependent ER Ca^2+^ release and altered ER–mitochondria contact sites. Future work will be required to determine which of these upstream Ca^2+^ sources predominantly contribute to mitochondrial overload in PDR-1-deficient muscle.

Skeletal muscle involvement in PD has traditionally been attributed to reduced physical activity secondary to dopaminergic degeneration. However, our findings in *C. elegans* suggest that impaired mitophagy and defective mitochondrial Ca^2+^ handling in mature muscle fibers may independently compromise mitochondrial quality and accelerate the decline in locomotor activity, in addition to neurological dysfunction. These results suggest that therapeutic strategies aimed at normalizing mitochondrial Ca^2+^ handling and reducing mtROS in skeletal muscles may warrant investigation as a strategy to preserve muscle mitochondrial function in PD. Consistent with this idea, a study using a zebrafish model of PD reported that gene inactivation of the mitochondrial calcium uniporter (*mcu*) to suppress excessive accumulation of mitochondrial Ca^2+^ exerted neuroprotective effects and improved the disease phenotype [42]. Our demonstration that *mcu-1* inhibition prevents mtROS elevation in PDR-1-deficient *C. elegans* muscle cells therefore supports the concept that targeting mitochondrial Ca^2+^ homeostasis may help preserve muscle function in PD.

Previous work has shown that age-associated increases in [Ca^2+^]_mito_ promote mitophagy and mitochondrial fragmentation in *C. elegans* body-wall muscle and that inhibition of MCU-1 suppresses these age-associated mitochondrial changes [10]. Consistent with these observations, early *mcu-1* RNAi suppressed mitochondrial fragmentation and preserved nuclear GFP fluorescence in WT animals in the present study. Given the role of PDR-1 in mitophagy, PDR-1 deficiency attenuated age-associated mitochondrial fragmentation, resulting in an apparently preserved mitochondrial morphology. Despite this apparently preserved morphology, *pdr-1(gk448)* animals exhibited loss of nuclear GFP fluorescence, consistent with nuclear degradation and muscle cell damage. Notably, early *mcu-1* RNAi preserved nuclear GFP fluorescence in *pdr-1(gk448)* animals, suggesting that MCU-1-dependent Ca^2+^ influx contributes to the nuclear phenotype associated with PDR-1 deficiency. In contrast, *mcu-1* RNAi initiated on day 7 of adulthood reduced [Ca^2+^]_mito_ and mtROS in *pdr-1(gk448)* animals but did not suppress nuclear degradation. These findings suggest that MCU-1-dependent Ca^2+^ influx acts at an earlier stage to promote mtROS accumulation and the mitochondrial and nuclear alterations associated with PDR-1 deficiency, and that inhibition of MCU-1 after cellular damage has progressed may be insufficient to reverse its structural consequences.

## Data Availability

The raw data supporting the conclusions of this article will be made available by the authors upon request.

## Supplementary Materials

The following supporting information can be downloaded at: https://www.mdpi.com/article/10.3390/antiox15081043/s1, Figure S1: Representative images of categorized mitochondria and nuclei. Figure S2: The *mcu-1* mutation does not alter the expression levels of *pdr-1* or *lgg-1*, and the *pdr-1* mutation does not alter *mcu-1* expression. Figure S3. Representative images of mitochondrial morphology and nuclear GFP fluorescence in body-wall muscle cells. Table S1: List of primers used in this study.

## Abbreviations

PD	Parkinson’s disease
MCI	Mitochondrial complex I
Ca^2+^	Calcium
MCU	mitochondrial Ca^2+^ uniporter
mtROS	Mitochondrial reactive oxygen species
RNAi	RNA interference
ΔΨm	Mitochondrial membrane potential
WT	Wild-type
NGM	nematode growth medium
VC	Vector control
[Ca^2+^]_mito_	Mitochondrial Ca^2+^ levels
OD600	Optical density at 600 nm
A.U.	Arbitrary units
TMRE	Tetramethylrhodamine ethyl ester
TCA	Tricarboxylic acid
IP_3_	1,4,5-trisphosphate
ER	Endoplasmic reticulum
