# Supplementary material for: Mitochondrial Ca2+ Influx via MCU-1 Contributes to Oxidative Mitochondrial Defects in PDR-1/Parkin-Deficient Caenorhabditis elegans Body-Wall Muscle
Source: Antioxidants (Basel). 2026 Aug 21;15(8):1043. doi: 10.3390/antiox15081043 (PMC13510160; doi:10.3390/antiox15081043)
Supplement: Supplementary file 1 [file antioxidants-15-01043-s001.zip › antioxidants-4380337-supplementary.pdf]

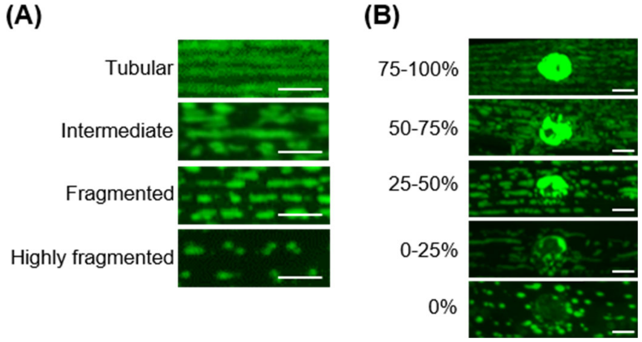

Figure S1. Representative images of categorized mitochondria and nuclei.
(A) Categorized mitochondria. (B) Categorized nuclei. Scale bars: 5 μm.

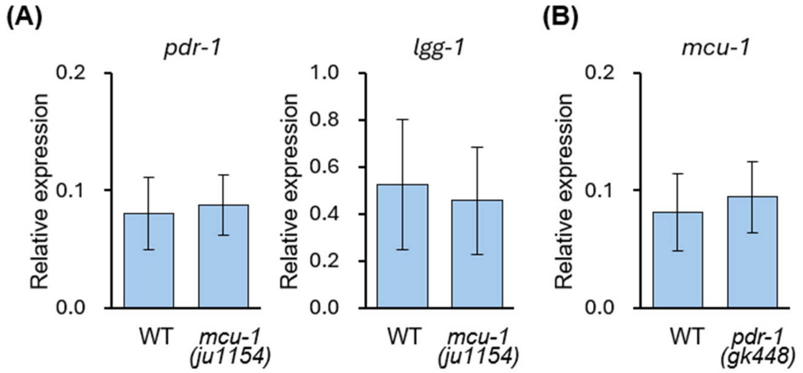

Figure S2. The *mcu-1* mutation does not alter the expression levels of *pdr-1* or *lgg-1*, and the *pdr-1* mutation does not alter *mcu-1* expression.

(A) Expression levels of *pdr-1* and *lgg-1* in WT and *mcu-1(ju1154).*

(B) Expression levels of *mcu-1* in WT and *pdr-1(gk448).*

The *act-1* gene was used as the internal standard. The graph shows the relative expression levels normalized to *act-1.* Data are means of six biological replicates. Error bars indicate SD.

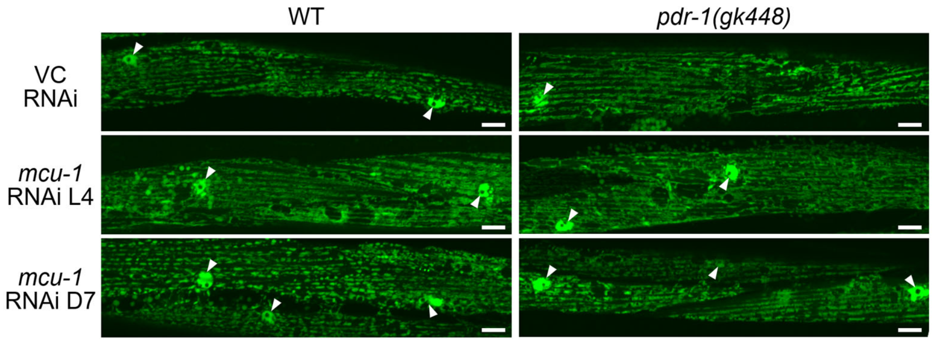

Figure S3. Representative images of mitochondrial morphology and nuclear GFP fluorescence in body-wall muscle cells.

Representative fluorescence images of body-wall muscle cells of WT and *pdr-1(gk448)* animals on day 10 of adulthood. These images correspond to the data shown in Fig. 6C,D. Animals were treated with VC RNAi, *mcu-1* RNAi initiated at the L4 stage, or *mcu-1* RNAi initiated on day 7 of adulthood. Images were obtained from transgenic *C. elegans* expressing mitochondrion-targeted GFP (mtGFP) and nucleus-targeted GFP (nucGFP) on day 10 of adulthood. Arrowheads indicate nuclei. Scale bars: 10 µm.

Table S1. List of primers used in this study.

Primer name	Sequences (5'-3')
pdr-1 RNAi Fw	TGGCGGCCGCTCTAGAGAAAAACAGGTCAACGTAGGAA
pdr-1 RNAi Rv	CGGTATCGATAAGCTAGCACTCGGTCATTTGCC
pdr-1 Fw	CAAGATGTGCACCATTTCCA
pdr-1 Rv	GTCATCATAGGGCTCCCAGA
lgg-1 Fw	CAATGTCAGACCATCACGAGGA
lgg-1 Rv	TACACATTCGTCGGCGGATA
mcu-1 Fw	CACAACAACAGCCTCCTCAA
mcu-1 Rv	GGCAAGGCTCATTTCTTGAC
act-1 Fw	CCTCTCCACCTTCCAACAGA
act-1 Rv	AGAAAGCTGGTGGTGACGAT
